# Measurement of Electrical Conductivity for a Biomass Fire

**DOI:** 10.3390/ijms9081416

**Published:** 2008-08-13

**Authors:** Kgakgamatso Mphale, Mal Heron

**Affiliations:** 1 Physics Department, University of Botswana, Private Bag 0022, Gaborone, Botswana; 2 Marine Geophysical Laboratory, James Cook University, Townsville, QLD 4811, Australia. E-Mail: Mal.Heron@jcu.edu.au (M. H.)

**Keywords:** biomass, forest fires, flashover, thermal ionization, alkalis

## Abstract

A controlled fire burner was constructed where various natural vegetation species could be used as fuel. The burner was equipped with thermocouples to measure fuel surface temperature and used as a cavity for microwaves with a laboratory quality 2-port vector network analyzer to determine electrical conductivity from S-parameters. Electrical conductivity for vegetation material flames is important for numerical prediction of flashover in high voltage power transmission faults research. Vegetation fires that burn under high voltage transmission lines reduce flashover voltage by increasing air electrical conductivity and temperature. Analyzer determined electrical conductivity ranged from 0.0058 - 0.0079 mho/m for a fire with a maximum temperature of 1240 K.

## 1. Introduction

When a weakly ionized medium is irradiated with electromagnetic energy, electrons in the medium gain a directed drift velocity in the direction opposite that of the applied electric field of the incident wave. The concentration of electrons, which can be up to 10^18^ m^−3^ in a vegetation fire [1] and ions which are in motion both contribute to the electrical conductivity of the weakly ionized fire. The contribution of the neutrals is in the energy dissipation processes in the fire, e.g., collisions with electrons. Vegetation fires are diffusion flames which are seeded with plants’ omnipresent alkali nutrients, more especially potassium and sodium salts. Electrical conductivities of flames seeded with alkalis have been determined, e.g., in Olson *et al*. [2] and Schneider *et al*. [3]. According to [2], radio frequency measured electrical conductivities of high temperature hydrogen-oxygen flames seeded with alkalis were in the range of 0.04–0.27 mho/m.

The experiment considers a moderate intensity vegetation fire. The fuel, which is natural vegetation, contains up to 3.4% potassium on dry weight basis [4] of which up to 20% are ionized [5]. X-band microwaves were caused to propagate in the vegetation fire to determine electrical conductivity from network analyzer measured S-parameters.

### 2. Ionization in Vegetation Fires

Combustion of vegetative matter creates a hot environment with temperatures in excess of 1200 K. The high temperature environment thermally excites incumbent flame particles. The energized particles become electronically unstable, to the extent that they lose their outer shell electrons on collision with other flame particles, a process that occurs on selective basis determined by temperature and ionization potential. This process is called thermal ionization. Potassium and graphitic carbon (*Cn*) are vegetation fire particles that are likely to produce appreciable ionization. This is due to the fact that the particles have low ionization energy and work functions of 4.34 and 4.35 eV respectively [6]. Thermal ionization of the excited flame species (FL*(g)) occurs by the following reaction equation:

(1) $${\text{FL}}^{*}(\text{g})\Leftrightarrow {\text{FL}}^{+}(\text{g})+{\text{e}}^{-}$$

Another process by which ionization may occur in the flame is chemi-ionisation. In chemi-ionisation, dissociation reactions provide part of the energy required for ionization since there are exothermic and the rest comes from the flame. Methyl radical is known for its contribution to flame chemi-ionisation reactions e.g., in Sorokin *et al.,* [6]. CH radical reacts with oxygen atoms in the flame to produce CHO^+^, a primary ion in hydrocarbon flames and electrons according to the following reaction equation:

(2) $${\text{CH}}^{*}(\text{g})+\text{O(g)}\Leftrightarrow {\text{CHO}}^{+}(\text{g})+{\text{e}}^{-}$$

### 2.1. Electrical Conductivity of the Fire

The complex electric conductivity of the weakly ionized fire given by the equation

(3) $$\sigma \;=\frac{{\text{Nq}}_{\text{e}}^{2}}{{\text{m}(\upsilon }_{\text{eff}}+\text{i}\omega )}=\sigma_{\text{r}}+\text{i}\sigma_{\text{i}}$$

where the real (σ_r_) and imaginary (σ_i_) parts are:

(4a) $$\sigma_{\text{r}}=\frac{\varepsilon_{0}\omega_{\text{p}}^{2}\upsilon_{\text{eff}}}{{(\upsilon }_{\text{eff}}^{2}{+\omega }^{2}\text{)}}$$

and

(4b) $$\sigma_{\text{i}}=\frac{\varepsilon_{0}\omega_{\text{p}}^{2}\omega }{{(\upsilon }_{\text{eff}}^{2}{+\omega }^{2}\text{)}}$$

where also in (4a) and(4b), $\omega_{\text{p}}={\left(\frac{{\text{Nq}}_{\text{e}}^{2}}{{\text{m}\varepsilon }_{0}}\right)}^{1/2}$, ɛ_0_ are plasma collision frequency and free space permittivity. The real and imaginary parts of electrical conductivity are related to dielectric permittivity of an ionized gas by the relation:

(5) $$\varepsilon_{\text{r}}=1+\frac{\sigma_{\text{r}}}{{\text{i}\omega \varepsilon }_{0}}+\frac{\sigma_{\text{i}}}{{\omega \varepsilon }_{0}}$$

Propagation constant (γ) of an electromagnetic wave traversing a weakly ionized medium such as fire is given by the relation:

(6) $$\gamma^{2}=-\mu_{0}\varepsilon_{0}\omega^{2}\varepsilon_{\text{r}}\Leftrightarrow \gamma \;=\text{i}\frac{\omega }{\text{c}}\sqrt{\varepsilon_{\text{r}}}$$

The γ is a measure of the rate of electromagnetic energy loss through the attenuation index ( α_f_ ) and wave dispersion through the refractive index (β_f_ ). It can also be given in terms of the indices as:

(7) $$\gamma \;=\alpha_{\text{f}}+\beta_{\text{f}}$$

When X-band microwaves illuminate weakly ionized, highly collisional atmospheric pressure flame plasma, α_f_ andβ_f_ are related to electron-neutral collision frequency and ionization by the expressions given by Mphale *et al.,* [9] as:

(8) $$\alpha_{\text{f}}≅\frac{\upsilon_{\text{eff}}}{2\text{c}}\left[\frac{\omega_{\text{p}}^{2}}{{(\omega }^{2}{+\upsilon }_{\text{eff}}^{2}\text{)}}\right]$$

and

(9) $$\beta_{\text{f}}≅\frac{\omega }{2c}\left[1+\frac{\omega_{\text{p}}^{\text{4}}}{8{\left(\omega^{2}{+\upsilon }_{\text{eff}}^{2}\right)}^{2}}\frac{\upsilon_{\text{eff}}^{2}}{\omega }\right]$$

where ω, c and υ_eff_ are propagation cyclic frequency, speed of light and effective electron-neutral collision frequency, respectively.

By comparison, equations (4a) and (8) give:

(10) $$\sigma_{\text{r}}=2\text{c}\varepsilon_{0}\alpha_{\text{f}}$$

Equations (4b) and (5) give:

(11) $$\sigma_{\text{i}}=\frac{\sigma_{\text{r}}(\varepsilon_{\text{r}}-1)}{\varepsilon_{\text{r}}}$$

The magnitude of the fire conductivity |σ| is given by the relation:

(12) $$|\sigma |\;={\left(\sigma_{\text{i}}^{2}+\sigma_{\text{r}}^{2}\right)}^{1/2}$$

## 3. Results and Discussion

### 3.1. Flame Temperatures

**Figure 1:**
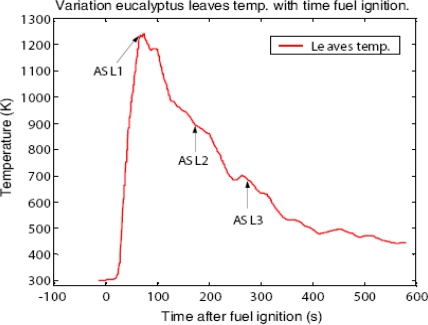
Variation of eucalyptus leaves surface temperature with time since ignition.

Flames as high as 75 cm were observed during the combustion. The eucalyptus leaves fire took 5 to 7 minutes to extinguish. During combustion flame temperatures at the fuel surface were recorded online by a computer and are shown in Figure 1.

The leaves’ surface temperature rose rapidly to reach a maximum of 1240 K in 74 s. The first set of S-parameters (at ASL1) was logged in at 68 s after ignition of the leaves. At ASL1, the temperature of the litter surface was 1235 K and corresponded to the time when eucalyptus leaves flame filled the whole inner volume of the burner. After reaching the climax, the surface temperature dropped rapidly, though not as quickly as it rose after ignition, to ASL2, a second login point where the eucalyptus flame was observed to fill the inner hollow of the burner. ASL2 is at 153 s after ignition time. At ASL2, the leaves surface temperature was observed to be 946 K. Several s-parameter were logged in since ASL2, those selected for analysis were those logged in at ASL3 which corresponded to 269 s since ignition. Surface temperature at ASL3 was 695 K.

### 3.2. Electrical Conductivity of the Fire

**Figure 2:**
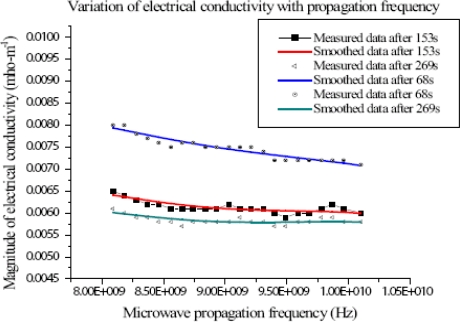
Variation of flame conductivity with propagation frequency at 68,153 and 269 s after ignition.

The conductivity of the fire at X-band frequencies was observed to be temperature dependent. At the time at which the flame surface temperature was the hottest, i.e., at ASL1, the conductivity was the highest. It steadily decreased with the increase in frequency from 0.0079 - 0.0071 mhom^−1^ for 8 - 10 GHz range, respectively (see Figure 2). At 153 s after ignition, thus, at ASL2, the magnitude of flame conductivity decreased with increase in propagation frequency. It decreased from 0.0064 - 0.0060 mhom^−1^ in the frequency range. This was at temperature 946 K. The decrease was not as rapid as at ASL1 (Figure 2).

At 269 s after ignition, i.e., at ASL3, the magnitude of flame conductivity also decreased with increase in propagation frequency. It decreased from 0.0060 - 0.0058 mhom^−1^ for the frequency range 8 - 10 GHz, respectively. This was at temperature of 695K.

The electrical conductivity values are low compared to those measured by other means because the flame temperatures were much lower than those cited in [2, 3]. Conductivity of flames is temperature dependent. Another possibility is that the fire may not have filled the whole combustion chamber, which could lead to microwave beam filling error. However, by visual inspection, those analyzed for propagation constant corresponded to situations where the fire filled the whole volume of the chamber.

## 4. Experimental Section

### 4.1. Network Analyzer and Burner System

**Figure 3:**
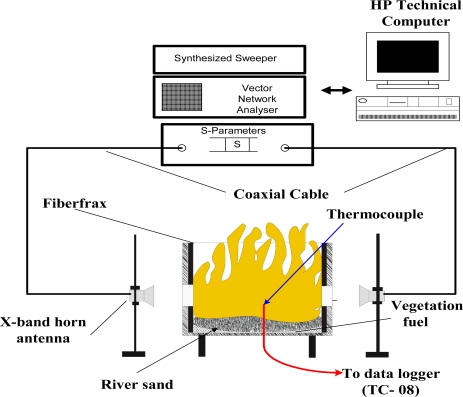
Network analyzer set up for S_21_ and S_11_ parameter measurements.

Dielectric permittivity and propagation constant of the fire was determined by using a Hewlett- Packard 8577C vector network analyzer. The analyzer was connected to a 733 MHz computer for logging S-parameter data (see Figure 3). A hexagonally shaped burner with an insulated wooden casing was used for the combustion of eucalyptus leaves. Inside the burner, a thick (8 cm wide) thermally insulating material known as Fiberfrax was used to protect wood from the fire and heat.

A combustion chamber, circular in cross-section was lined with this material. Two vent holes of 25 mm diameter were drilled on each of the sides, except for the sides with horn inlets, to allow air to enter and mix with fuel during combustion. Two holes of horn dimensions were also cut out from the burner casing directly opposite to each other and wooden supports were provided to secure the horns firmly to the wooden casing. The internal diameter of the burner was lined up with Fiberfrax and was set to 50 cm. Two x-band transmit-receive horns were used in the experiment. They were connected to a network analyzer through the two-port s-parameter test set by coaxial cables. High quality mode transition adapters were used to make the connections between coaxial cables and the horns. Eucalyptus leaves combusted in the burner were of fuel load 6.92 ton/ha and were collected fifteen (15) days before the experiment so that they could to dry in a laboratory to maximize combustion efficiency during burning.

### 4.2. Flame Temperature Measurement

A thermocouple used to measure surface temperature in the burner. It was cut from a double braided fiberglass insulated chromel-alumel (24-G/G) thermocouple wire 50 μm in diameter. The thermocouple wire had a fiber glass shield which can withstand temperatures up to 450 °C. The type K thermocouple wires were electro-fused at one end to make perfect junction (bead). The bead was made small (∼ 0.12 mm) to minimize error in temperature readings due to heat transfer processes, e.g., radiation. The thermocouple was tested for accuracy and consistency in produced thermoelectric voltage using a hot air gun, thermometer and multimeter. The thermocouple was inserted from the bottom of the burner with the electro-fused junction left protruded 1cm above the fuel surface into the fire. The thermocouple was then connected to a PICO® Tech TC-O8 data logger and a laptop for online temperature measurement (see Figure 3).

### 4.3. S-Parameter Measurements

The 8577C network analyzer set is designed to sweep from 50 MHz to 40 GHz and logging in 601 S-parameter data points in each and every sweep. However, for the experiment, frequency range of interest was between 8 and 10 GHz. The data are then uploaded and analyzed by the computer. The analyzer takes 2 s to sample over one sweep, and then there is a latency of about 50 s before the next sweep can be initiated. The network analyzer was calibrated before use. The calibration method used in the experiment is the Transmit-Reflect-Line (TRL). Varadan et al. [7] give a full account of calibrating a network analyzer using TRL method. Several sweeps and logging of s-parameters were carried, but those in which flames filled the entire internal hollow space of the burner were chosen for s-parameter analysis. The selected logged in S-parameters were those at ASL1, ASL2 and ASL3, which were at 68,153 and 269 s since ignition.

### 4.4. Determination of Propagation and Dielectric Constants from S-parameters

The network analyzer measures the scattering parameters (S-parameters) from which the propagation and dielectric constants can be calculated. From the S-parameters analysis, propagation constant is related to the propagation factor (T) by the relation:

(13) γ =[ln(1/T)]/d

Kadaba [8] gives a full account of calculating T and complex dielectric permittivity from S-parameters. Generally, dielectric permittivity is given as:

(14) $$\tilde{\varepsilon }\;=\;\frac{\lambda_{0}}{\mu }{\left(\frac{\text{i}}{2\text{d}\pi }\text{ln}(\text{T})\right)}^{2}$$

where $\mu \;=\;\lambda_{0}\left\{\left(\frac{\text{i}}{2\text{d}\pi }\text{ln}(\text{T})\right)\left(\frac{1+\Gamma }{1-\Gamma }\right)\right\}$ and λ_0_ is free space wavelength.

Then attenuation ( α_f_ ) and refractive (β*_f_* ) coefficients are determined from (7) as they are real and the imaginary parts of propagation constant (γ). Complex dielectric permittivity ( ɛ̃ ) of the fire is then calculated from relation (14).

### 4.5. Determination of Flame Electrical Conductivity

The real and imaginary parts of electrical conductivity of the eucalyptus fire were measured at three different times (i.e., from ASL1 to ASL3) during combustion. They were determined from S-parameters using Equations (13) and (14).

## 5. Conclusions

Microwave electrical conductivity of eucalyptus fire with maximum temperature of 1240 K ranged from 0.0058 – 0.0079 mhom^−1^. The electrical conductivity could have been higher for very high intensity vegetation fires, as it is temperature and ionization dependent. Comparatively, the measured conductivity was far much lower than that reported for hydrogen-oxygen flames in [2]. Hydrogen-oxygen flames are usually at a very high temperature. When seeded with alkalis, as it was the case with the flames in [2], their electrical conductivity is increased. In [2], the alkali seeded hydrogen-oxygen flames conductivity was measured at the frequency of 20 MHz and was in the range of 0.04-0.27 mhom^−1^.
